# On Diseases of Infancy; Their Importance and Fatality

**Published:** 1855-09

**Authors:** Gunning S. Bedford

**Affiliations:** Professor of Obstetrics, &c., &c., University of New-York


### SELECTIONS.

*On the Diseases of Infancy; their Importance and Fatality.* By GUNNING S. BEDFORD, M. D.,* Professor of Obstetrics, &c., &c., University of New-York.

GENTLEMEN:—You have had before you during the present session of lectures a great variety of infantile diseases; you have observed the maladies peculiar to the new born infant, and have not failed to contrast them with those which develop themselves at a later period of childhood. In the study of the diseases of infancy, there is a peculiar interest; and if no other motive should urge the physician to a faithful investigation of these affections, philanthropy alone, it appears to me, presents irresistible claims. The bills of mortality exhibit a fearful picture, and while they are humiliating to our science, they should prompt an earnest endeavor to check this melancholy outlet to human life. If we are to credit statistical tables, gathered with great care, and with a definite object, one-fourth of the children born in France die before they have completed their first year! To the philosopher, to him who reasons, is fond of demonstration, and wishes data for his opinions, the following question in connection with the above results, will very naturally present itself: Is this fatality in infancy unavoidable, and beyond the limits of science? It becomes us to examine this question; it stands at the very foundation of the topic now under discussion, and exhibits for the contemplation of the physician, subjects of the deepest interest. I assume the negaiive side of this question. It can, I think, be demonstrated that the mortality of early life is due not to necessity, but to various causes which, measurably at least, are within control.

*Being an extract from Professor Bedford's new work on "The Diseases of Women and Children," which will be published during the present summer by Messrs. S. S. & W. Wood, 261 Pearl St. New-York.

It is unfortunate that authors, and also teachers, in their discussion of infantile diseases, have described them too much in the abstract. Take, for example, most of the treatises on this subject, and what do you find? A given affection is spoken of its causes, symptoms, diagnosis, prognosis, pathology, and treatment are minutely discussed : but the principal point is passed over in silence, the point most material for the physician to remember, and without which he can have no basis of hope that his treatment will prove curative. The point to which I allude is this—*that the diseases of infancy differ from those of the adult as do the structure and physiology of the one from those of the other ; there is simply an analogy, nothing more.* With few exceptions, the error of which I speak, pervades the works put into your hands as guides for the treatment of the maladies incident to early childhood; you go forth on your mission of duty with false principles, and, as a necessary consequence, in your conflict with diseases defeat will be your portion. The true requisite for the physician, if he desire to treat successfully the diseases of infancy. is to understand the peculiarities of that tender age; he must examine and study with no ordinary attention the characteristics of structure, and his mind must become familiar with its special physiology. A work on the physiology and pathology of infancy, with a direct reference to the differences of healthy and morbid action as it exists in the young and adult subject, is what at this time is much needed; it would shed fresh light on one of the most interesting departments of the profession, and would lead to a salutary influence in our application of therapeutic agents.

The new-born infant is altogether a different being from the adult; the mechanism of the one is imperfect, while that of the other is complete and perfect in all its parts. The one is engaged, if I may so speak, in the work of development, while the other, whose development is achieved, is occupied with the repair of the waste to which its organs are constantly subjected. In the infant, the nutritive functions, through which the general fabric is completed, are in full activity—organic life, indeed, is here so exclusive that it may be said with truth, that in the earlier periods the infant enjoys but one existence—the animal functions are yet in slumber, the intellectual faculties undeveloped. Ratiocination is not one of the attributes of the new-born child, nor does it enjoy the power of the locomotion. Both these latter are but results of healthy development, the former of the brain, the latter of the bones, muscles, and nerves. From the moment of birth, nature becomes actively engaged in perfecting the various organs of the infant; this work of development is necessarily rapid, and the constant and hurried transitions through which the child is passing are not only fruitful cases of disturbed action, but require a special and guarded therapeutics. The young infant possesses no language of the tongue to tell its sufferings, hence the difficulty of the physician oftentimes to detect the true nature of the disease. Conjecture is thus frequently substituted for positive knowledge, and conclusions hastily arrived at, not only unjustified, but too often fatal. Though the infant can not speak, yet it possesses a language perfectlly intelligible to the accurate observer—it is the language of expression. Some one has said, and most truly so, "that the countenance of the young child is the mirror of nature." Yes, gentlemen, it is a faithful reflex—its smile is that of pleasure and sincerity, while the indication of pain is but the offspring of suffering. Its countenance knows not the guile of the hypocrite—its expression is that of truth, and hence in health, under the influence of physical quietude, every feature bears the impress of tranquility.

Billard and Jadelot in France, and Underwood in England, have given great attention to this subject—they have studied carefully the countenance in health and disease—the eye, the mouth, the nose, the cry, the respiration, the gestures, the attitude—in a word, the *tout ensemble* of expression, has constituted for them a subject of profound reflection—and their varied and constant opportunities for observation, have led to important results. Bouchut, in his *Traite pratique des Maladies des Nouveaux-nes,* has elaborated this subject, and you can refer to his able work with much profit. Hippocrates has drawn particular attention to the change of physiognomy in the different diseases of the adult, and in this he has been followed by some of his successors. Little, however, has been said with regard to these changes in the infant—and it has been left for the moderns, our own cotemporaries, to deduce practical and important inferences respecting morbid action in the infant, based upon the peculiar expression of countenance.

This is a topic worthy of your consideration. I have on various occasions called your attention to it in connection with the numerous infantile diseases which have been presented at the Clinique—and I shall continue to do so, for I regard a knowledge of this language of expression as one of the indispensible elements of success in the management of the maladies peculiar to infancy. But in what way is the knowledge to be obtained? Exclusively by observation. All that is valuable in the practical part of your science is the result of observation. Simple hypothesis is simple conjecture, but when tested and proved to be true by repeated observation, it then becomes a reality; it loses its hypothetical character. and is accepted as a *fact.* So, too, with the language of expression as a means of diagnosis.

You have already seen that the first year of existence is one of alarming fatality—and I am disposed to believe that of all the causes which conspire to this early destruction of human life, there are two peculiarly constant and unerring in their effects—I mean *improper food* and *over-drugging.* If you will consult your note-books, they will tell you of the numerous cases of emaciation from diarrhœa which have been presented at the Clinique almost in the last stages of decay, and which were traced to food which the infant could not assimilate—the food, consequently, became a source of irritation to the muco-intestinal surface, keeping up frequent and profuse discharges, involving the entire system in disturbed action, and ultimately leading to death. Count the multitudes of young children swept from earth by what the bills of mortality denominate "*cholera infantum,*" and you will then be enabled to approximate to some idea of the fatal effects of food unfitted to the frail and sensitive organs of the infant! Nature has abundantly provided for the nourishment and development of the fœtus during its sojourn in its mother's womb; and, after its birth, that same nature, always vigilant, and governed in her actions by a conservative principle, has also provided a nutriment suited to its wants and physical capacity. Under ordinary circumstances, if the infant be permitted to take this nutriment thus prepared, and of such easy elaboration, it will be found to thrive, and pass with much greater certainty through the period of life usually so fatal to it. But, unfortunately, nature has to contend with many rivals in the persons of *experienced* nurses, and occasionally officious physicians. The infant has scarcely come into the world, certainly not longer than to be washed and dressed, before its little stomach is made the receptacle either of medicine, which it was never intended it should take, or various compounds, such as teas, ptisans, panadas, etc., and on the sole ground that the "poor little dear" must be purged, or that it is hungry. If it should cry, then the evidence of hunger is beyond all doubt! This is all wrong. It is a pernicious practice, and one which I trust will never meet your sanction.

There is a striking analogy in the laws instituted for the regulation of the health of man, and those which obtain in the health of animals. Instinct affords you very strong, I might say irresistible, evidence that nature, when not interfered with, is quite adequate, while disease does not exist, to provide for the internal wants of the new-born child. Are any of you fond of the canine species? If so, how often must you have observed the little pup soon after its birth—look at that pup and see how true it is to the impulses of nature! It is scarcely in the world before it seeks the teat of its mother. It draws *ad libitum* upon that fountain to which it has a birth-right, and from which it extracts the elements, not only of nutrition, but of health. No medicine or artificial food given here, and consequently none of those derangements, the immediate result of officiousness. And why is this? Simply because where instivct prevails, nature exercises a sovereign control, and exhibits in full beauty her power and perfection. Man boasts of his reason, but oftentimes, through his own perversion of it, he finds that in many of its operations it is less than instinct! I leave you to reconcile the paradox—all experience proves that my remarks are just and susceptible of demonstration in a thousand different ways.—*Virginia Medical and Surgical Journal.*

